# The Effect of a Pilot Pediatric In-Patient Department-Based Smoking Cessation Intervention on Parental Smoking and Children’s Secondhand Smoke (SHS) Exposure in Guangxi, China

**DOI:** 10.3390/ijerph13111109

**Published:** 2016-11-08

**Authors:** Kaiyong Huang, Li Yang, Jonathan P. Winickoff, Jing Liao, Guangmin Nong, Zhiyong Zhang, Xia Liang, Gang Liang, Abu S. Abdullah

**Affiliations:** 1 School of Public Health, Guangxi Medical University, Nanning 530021, Guangxi, China; huangka0319@sina.com (K.H.); yangli8290@hotmail.com (L.Y.); rpazz@163.com (Z.Z.); 2 MGH Center for Child and Adolescent Health Research and Policy, Harvard Medical School, Boston, MA 02115, USA; jwinickoff@partners.org; 3 Department of Pediatrics, The First Affiliated Hospital of Guangxi Medical University, Nanning 530021, Guangxi, China; gxlmd@126.com (J.L.); ngm8525@hotmail.com (G.N.); 4 Foreign Language School, Guangxi Medical University, Nanning 530021, Guangxi, China; xialiang17@gmail.com; 5 Pharmaceutical School, Guangxi Medical University, Nanning 530021, Guangxi, China; 6 Global Health Program, Duke Kunshan University, Kunshan 215316, Jiangsu, China; 7 Duke Global Health Institute, Duke University, Durham, NC 27708, USA; 8 Boston University School of Medicine, Boston Medical Center, Boston, MA 02118, USA

**Keywords:** secondhand smoke, exposure, smoking cessation intervention

## Abstract

Children’s exposure to secondhand smoke (SHS) at home has numerous adverse health effects. This study evaluated the effects of a pediatric in-patient department-based pilot smoking cessation intervention for household members to reduce children’s SHS exposure and encourage smoking cessation. A pre-post test design study was designed to assess the effectiveness of a telephone counseling intervention on household members of hospitalized children in pediatric departments. Data were collected with a standardized Chinese language questionnaire. At the three-month follow-up survey, the proportions of household members who reported adopting complete smoking restriction at home (55%), did not smoke at home at all (37%), did not allow others to smoke in the car (70%), or did not allow others to smoke around the child (57%) were significantly higher than the self-reported responses at the baseline survey. The proportions of household members who reported smoking at home (49%) and in the car (22%) were significantly lower than the baseline survey. Overall, 7% of the participants had reported quitting smoking after three months. Pediatric in-patient department-based telephone counseling for smoking cessation was found to be acceptable to Chinese parents. The intervention encouraged few parents to quit smoking, but encouraged more parents to take measures to reduce children’s SHS exposure.

## 1. Introduction

Secondhand smoke (SHS; also known as environmental tobacco smoke) exposure in children contributes significantly to morbidity and mortality [1]. It is reported that children exposed to SHS have higher rates of respiratory illness, asthma and asthma exacerbations, cough, middle ear infections, hospitalizations, childhood cancer, cardiovascular disease, and sudden infant death syndrome [2,3,4]. Worldwide, 40% of children, 33% of male non-smokers, and 35% of female non-smokers were exposed to SHS in 2004 [3]. During this period, an estimated number of 603,000 deaths were attributable to SHS, 28% of which occurred in children [3]. China is the largest producer and consumer of tobacco in the world with 350 million smokers [5], and has almost 740 million non-smokers passively exposed to SHS, including 180 million children under 15 years old [6]. The magnitude of health problems caused by SHS underscore the urgent need for targeted interventions, especially in this country.

Establishing smoke-free policies at work and in public places and creating smoke-free homes are effective interventions for reducing SHS exposure [7]. A growing awareness of the dangers of SHS has led to increased efforts to establish smoke-free policies to eliminate SHS exposure in public places and workplaces [7,8,9]. However, the primary location for SHS exposure for children is in the home [10]. The proximity, intensity, and duration of SHS exposure at home are often greater than those in public settings [11]. The 2010 China Global Adult Tobacco Survey (GATS) reported that the prevalence of SHS exposure at home (67%) was higher than that in workplaces (63%), government buildings (58%), and schools (37%) [12]. Strict home smoking bans contribute to preventing and reducing children’s exposure to SHS and increasing parental smoking cessation [13,14]. Moreover, smoking cessation is a priority for reducing the harms and burden caused by smoking attributable diseases [15,16].

For increasing smoking cessation and reducing SHS exposure, many intervention measures are available, including Quitline and face-to-face counseling [17], internet- and text-message-based interventions [18,19], and smoking cessation medications [20,21]. Pediatricians have a unique and important role to play in the protection of pediatric patients from the harmful effects of SHS, and the encouragement of smoking cessation among households [22,23]. The American Academy of Pediatrics and the US Surgeon General encourage the pediatricians to deliver interventions to promote household smoking cessation [24]. An earlier study showed that pediatricians providing smoking cessation advice to parents managed to help household members to quit smoking and reduce children’s SHS exposure [25]. Nevertheless, in China, few studies have examined the effect of pediatric setting-based delivery of smoking cessation interventions among household members. This study reports three-month follow-up results of a smoking cessation intervention for household members to reduce children’s SHS exposure in Guangxi, China.

## 2. Methods

### 2.1. Design and Sample

A pre–post test design study was conducted from November 2013 to May 2014 to assess the effect of a newly developed pilot smoking cessation intervention delivered over the telephone.

Subjects were recruited from the pediatric in-patient departments of the First Affiliated Hospital of Guangxi Medical University, the largest grade-three hospital in Nanning, Guangxi province. All members of the household of the children in the pediatric in-patient departments of the hospital, admitted for at least 24 h, were eligible to participate in the study. Hospital systems in China follow a grading system. The higher the grade, the larger the hospital and the more sophisticated the facility is. Grade-three hospitals are general or comprehensive hospitals at national, provincial, or city level (>500 beds) [22]. The hospital was conveniently selected as the study site.

Of 163 smoking households in the study, 126 smokers from 126 households (i.e., only one smoker per household; in case there was more than one smoker in a household, we recruited the smoker who smoked more cigarettes at home) provided telephone numbers to receive calls from smoking cessation counselors, but only 107 smoking households completed the baseline survey and voluntarily participated in the follow-up survey. 87 participants (81%) were successfully followed up on after 3 months.

### 2.2. Intervention

The intervention developed was telephone-based smoking cessation counseling and delivered by two junior pediatricians trained as smoking cessation counselors. Before the intervention delivery, four pediatricians in the hospital participated in a three-day (about 12 h) training session about smoking cessation intervention and passed an examination at the end of the training. The training consisted of lectures, demonstrations, case reviews, in-class discussion, and role-plays. The primary contents of the training included epidemiology of smoking and SHS exposure in China, health hazards of smoking and SHS exposure, strategies for smoking cessation including the use of cessation medications, and ethical aspects of human research. Once the pediatricians had a good command of the training contents and skill of intervention, smoking cessation intervention work would be carried out. Of the four pediatricians trained, only two were engaged in the delivery of telephone-based intervention.

The intervention was delivered over the phone at 3 different contacts after the baseline survey: Week 1 (30 min), Week 4 (10–15 min), and Week 8 (10–15 min). The intervention was based on the 5 As of the Public Health Service Clinical Practice Guideline for Treatment of Tobacco Dependence [26], highlighting the hazards of SHS on children’s health and the importance and strategies of quitting smoking. The smoking cessation intervention focused on the following aspects: (1) health risks of smoking and SHS exposure; (2) enforcing a strict no-smoking policy at home and in the car; (3) introducing common methods and medications for smoking cessation; (4) offering cessation brochures describing the health risks of smoking and children SHS exposure; and (5) providing a poster that reads, “Dad, the smell of your smoke makes me uncomfortable”, no-smoking signs, and stickers that read, “Don’t smoke if you love your child” and “Quit smoking for the well-being of your child’s health”, to serve as cues for reducing their child’s exposure to SHS at the end of the counseling [27].

### 2.3. Methods of Data Collection and Measures

Trained research assistants collected data at baseline and at follow-up. Firstly, interviewers visited each hospitalized child’s household members to explain the study and invite them to participate in it. Then, voluntary participants signed the consent form and completed the baseline survey in a face-to-face interview. Baseline interviews were held in a conveniently located room in the hospital for the household members and took about 50 min. The three-month follow-up survey was carried out by the same interviewer via phone call. The interviewer was not engaged with the delivery of counseling. Only those household members who agreed to be contacted at follow-up were contacted. Checks were carried out to ensure all questions had been answered; in case of any discrepancies (unclear answers, unfinished questions, logistic errors, or any combination of these), the investigators contacted the individual by telephone or requested a re-interview when necessary. As a token of appreciation, each participant received a small gift [28]. The study protocols were approved by the Ethical Committee of Guangxi Medical University (Number: 2013/ethics/SPH/03; date: 15 March 2013), and informed consent was received from all individuals who agreed to participate in the study.

We collected data with a standardized Chinese language questionnaire developed with reference to questionnaires previously used and validated by the investigator’s team in China [11,28]. The baseline questionnaire included questions on the following domains: (1) demographic characteristics; (2) household members’ smoking and quitting situation; (3) child’s health status; (4) frequency of children’s SHS exposure at home and in the car; and (5) knowledge and attitudes on smoking and SHS. The follow-up questionnaire included questions on the following domains: (1) children’s health status in the past three months; (2) household members’ smoking and quitting situation; (3) household members’ smoking practices in the past three months; (4) reduction in children’s household SHS exposure; and (5) actions taken by households to reduce children’s SHS exposure. Participants were also asked about the helpfulness of the intervention, the follow-up contacts, and the self-help materials [28].

### 2.4. Analyses

We coded all the questionnaires, entered all data in Epidata 3.1 (The EpiData Association, Odense, Denmark), and then carried out data consistency checks. To examine differences between the baseline survey and the three-month follow-up survey, a chi-square test was performed. A *p* value < 0.05 (two-tailed) was considered statistically significant. We analyzed the data with SPSS for Windows, version 13.0 (IBM, New York, NY, USA).

## 3. Results

### 3.1. Demographic and Other Characteristic Information

In the study, 107 household members voluntarily participated in the baseline survey, and 87 participants (81%) were successfully followed up after three months. No differences in characteristics were found between the baseline survey and the three-month follow-up survey, except for the viewpoint of “Important to adopt a no-smoking policy at home”. We found that 83% of household members thought it was important to adopt a no-smoking policy at home, which was significantly higher at the three-month follow-up than at the baseline survey (*p* < 0.05) (Table 1).

### 3.2. Effect of Smoking Cessation Intervention

Compared to the baseline, at the three-month follow-up, a significantly (*p* < 0.05) higher proportion of household members reported adopting complete smoking restriction at home (35% vs. 55%), reported that fewer smokers smoked at home (69% vs. 49%) or in the car (43% vs. 22%), did not smoke at home at all (37% vs. 21%), and did not allow others to smoke near the child (57% vs. 40%) or in the car (72% vs. 40%). However, we did not find significant differences in the number of participants that were thinking of quitting smoking (*p* = 0.687) and that ceased smoking for at least 24 h at least once (*p* = 0.848) between the baseline survey and the three-month follow-up survey (Table 2). Moreover, 7% (6/87) of household members had quit smoking (i.e., did not smoke any cigarette during the previous 7 days of the follow-up interview). 

## 4. Discussion

This study is among the first to describe the effect of a telephone-based smoking cessation intervention for members of children’s households to reduce children’s SHS exposure in China. The self-reported positive changes among the smoker households at the three-month follow-up to adopt complete smoking restriction at home suggests that the smoking cessation intervention may have played a positive role in reducing children’s exposure to SHS. The same result was also found in a randomized controlled trial in Shanghai, China [28], which reported that smoking hygiene intervention was effective in reducing children’s exposure to secondhand smoke. In the three-month follow-up survey, 57% of participants did not allow others to smoke around the children, and more than one-third did not smoke at home at all, with significant differences from the baseline survey. This might be because household members were conscious of the risks of smoking and SHS to children after accepting the smoking cessation intervention. However, for politeness and respect, which is part of Chinese cultural norms, it would be embarrassing to ask a smoking guest not to smoke in the house. Some smokers did not smoke in front of a child while at home, but probably would do so when outside.

We also found that a much higher percentage of participants at the three-month follow-up reported that they did not allow others to smoke in the car compared with the baseline survey. However, in the three-month follow-up survey, 22% of the participants reported that someone still smoked in their cars in the last three months, which was significantly lower than the baseline survey. This might be due to the fact that some smokers think that smoking in the car is not a serious problem if they open the window, even when children are there [29]. We found that the intervention did not have a great impact in helping household members to quit smoking. At the three-month follow-up, only 9% of participants stopped smoking for 24 h at least once, and 7% had quit smoking already. However, our findings differ from another study in the US that reported that the rates of ever achieving 24 h smoking cessation at twelve months were 57% in the intervention group and 60% in the control group. However, the rates of smoking cessation at three and twelve months were twice as great in the intervention group as in the control group (7.7% vs. 3.4% and 13.5% vs. 6.9%, respectively), and the difference was statistically significant at the twelve-month follow-up but not significant at the three-month follow-up [30]. The quitting rates achieved in our study were consistent with findings from other studies evaluating smoking cessation interventions with young women in community health centers [28], pediatric clinics [30], prenatal clinics [31], Planned Parenthood establishments [32], and pediatric settings [33]. It is suggested that the intervention components helped participants succeed in stopping smoking for 24 h at least once, and we should follow up for a longer time (i.e., six months or twelve months) to assess the effects of smoking cessation intervention in the future. The content and frequency of smoking cessation counseling that we provided also need to be evaluated for their effectiveness.

The following limitations should be noted. First, we recruited parents of hospitalized children from the largest grade-three hospitals in Guangxi, which may not represent the characteristics of participating parents attending other lower grade hospitals, as well as the non-hospitalized population. Second, the participants’ smoking history, as well as other factors, was ascertained based on self-reporting methods without any biochemical validation, which may have caused information bias. Third, not all participants were available for follow-up. However, we did not find any difference in the characteristics of those who were available and those who were lost for follow-up. Fourth, due to the self-reported nature of the data, there is no way to know for sure if the intervention changed household members’ smoking behavior and no current way to determine any actual changes to SHS exposure for the children. Future studies should consider using air nicotine monitors at home to assess the level of nicotine in ambient air or in children’s hair to assess exposure to SHS [34].

## 5. Conclusions

The pilot telephone-based smoking cessation intervention that we developed and delivered to the parents of children recruited from hospital in-patient departments was found to be acceptable to parents. The intervention encouraged few parents to quit smoking, but more parents were encouraged to take measures to reduce children’s SHS exposure. These findings should guide the development of future smoking cessation programs to be delivered through pediatric settings in China and other low- and middle-income countries.

## Figures and Tables

**Table 1:** Characteristics of the studied sample at the baseline and three-month follow-up surveys.

Variables	Baseline Survey (*n* = 107), *n* (%)	Three-Month Follow-up Survey (*n* = 87), *n* (%)	χ^2^	*p* Value
**Gender**				
Male	66 (62)	51 (59)	0.188	0.665
Female	41 (38)	36 (41)		
**Age**				
18–30	45 (42)	32 (37)	0.582	0.747
31–44	42 (39)	38 (44)		
Above 45	20 (19)	17 (19)		
**Ethnicity**				
Han	82 (77)	73 (84)	1.580	0.209
Other ethnicities	25 (23)	14 (16)		
**Education**				
Under high school	18 (17)	18 (21)	0.515	0.773
High school graduate	36 (34)	29 (33)		
College or above	53 (49)	40 (46)		
**Income**				
Below 3,000 CNY per month	27 (25)	18 (21)	0.602	0.740
3,000–6,000 CNY per month	52 (49)	46 (53)		
Above 6,000 CNY per month	28 (26)	23 (26)		
**Child’s age**				
Under 3 years	41 (38)	35 (40)	1.038	0.595
3–5 years	44 (41)	39 (45)		
Above 5 years	22 (21)	13 (15)		
**Child’s gender**				
Male	51 (48)	46 (53)	0.521	0.470
Female	56 (52)	41 (47)		
**Relationship to child**				
Father or mother	90 (84)	70 (80)	0.443	0.506
Other caregiver	17 (16)	17 (20)		
**Number of smokers at home**				
One	88 (82)	68 (78)	0.508	0.476
Two or more	19 (18)	19 (22)		
**Important to adopt no-smoking policy at home**				
Agree/strongly agree	66 (62)	73 (84)	11.669	0.001
Disagree/strongly disagree	41 (38)	14 (16)		
**Difficulty in adopting no-smoking policy at home**				
Agree/strongly agree	91 (85)	72 (83)	0.187	0.665
Disagree/strongly disagree	16 (15)	15 (17)		
**Confidence to not allow others to smoke in the house**				
Very/Somewhat confident	51 (48)	45 (52)	0.317	0.574
Not at all confident	56 (52)	42 (48)		

**Table 2:** Smoking status, quitting status, and secondhand smoke exposure reduction practices of the studied sample at the baseline and three-month follow-up surveys.

Variables	Baseline Survey (*n* = 107), *n* (%)	Three-Month Follow-up Survey (*n* = 87), *n* (%)	χ^2^	*p* Value
**Adopted complete smoking restriction at home**				
Yes	37 (35)	48 (55)	8.266	0.004
No	70 (65)	39 (45)		
**Thinking of quitting smoking in the next 6 months**				
Yes	7 (6)	7 (8)	0.162	0.687
No	100 (94)	80 (92)		
**Someone smoked at home in the last 3 months**				
Yes	74 (69)	43 (49)	7.806	0.005
No	33 (31)	44 (51)		
**Household members did not smoke at home at all**				
Yes	23 (21)	32 (37)	5.520	0.019
No	84 (79)	55 (63)		
**Did not allow others to smoke around the child**				
Yes	43 (40)	50 (57)	5.744	0.017
No	64 (60)	37 (43)		
**Someone smoked in the car in the last 3 months** *				
Yes	31 (43)	15 (22)	6.695	0.010
No	41 (57)	52 (78)		
**Did not allow others to smoke in the car** *				
Yes	30 (42)	47 (70)	11.395	0.001
No	42 (58)	20 (30)		
**Stopped smoking at least once—for at least 24 h—during 3 months before the follow-up** **survey**				
Yes	9 (8)	8 (9)	0.037	0.848
No	98 (92)	79 (91)		
**Had quit smoking** (did not smoke any cigarette during the previous 7 days of the follow-up interview)	Not applicable	6 (7)	-	-

* Totals may not equal to 107 or 87 because 35 subjects in the baseline survey and 20 in the three-month follow-up survey did not have a car.
